# Influence of enhanced nutrition and psychosocial stimulation in early childhood on cognitive functioning and psychological well-being in Guatemalan adults

**DOI:** 10.1016/j.socscimed.2021.113810

**Published:** 2021-04

**Authors:** María J. Ramírez-Luzuriaga, Ann M. DiGirolamo, Reynaldo Martorell, Manuel Ramírez-Zea, Rachel Waford, Aryeh D. Stein

**Affiliations:** a Nutrition and Health Science Program, Laney Graduate School, Emory University, 201 Dowman Drive, Atlanta, GA, 30322, USA; b Georgia Health Policy Center, Georgia State University, 55 Park Place NE, Eighth Floor, Park Pl NE, Atlanta, GA, 30303, USA; c Hubert Department of Global Health, Rollins School of Public Health, Emory University, 201 Dowman Drive, Atlanta, GA, 30322, USA; d INCAP Research Center for the Prevention of Chronic Diseases (CIIPEC), Institute of Nutrition of Central America and Panama (INCAP), Calzada Roosevelt 6-25 zona 11, Guatemala City, Guatemala

**Keywords:** Nutrition interventions, Psychosocial stimulation, Cognitive ability, Executive function, Psychological well-being, Structural equation modeling

## Abstract

**Rationale:**

Early-life nutrition interventions in low and middle-income countries have demonstrated long-term benefits on cognitive skills, however, their influence on socioemotional outcomes has not been fully explored. Moreover, the mediating processes through which nutrition intervention effects operate and are maintained over time are understudied.

**Methods:**

We followed-up a cohort of Guatemalan adults who participated as children in a community randomized food-supplementation trial. We examined associations of exposure to nutritional supplementation from conception to age 2 years with executive function (measured using three sub-tests of the NIH Toolbox Cognition Battery) and psychological well-being (measured using two sub-scales of the NIH Toolbox Emotion Battery) at ages 40–57 years (n = 1268). We used structural equation modeling to investigate the mediating role of psychosocial stimulation (measured in childhood using parent reports and ratings of home environments), cognitive ability (measured at ages 26–42 years using standardized tests), and executive function on the association of early-life exposure to nutritional supplementation with adult psychological well-being (*n* = 1640).

**Results:**

We found positive but inconsistent associations of nutritional supplementation in childhood with executive function and psychological well-being in adulthood. Psychosocial stimulation, cognitive ability, and executive function did not mediate the association of early-life nutritional supplementation with adult psychological well-being. We found strong and positive associations of psychosocial stimulation in childhood with cognitive ability, executive function, and psychological well-being in adulthood. Moreover, we observed no interaction of exposure to nutritional supplementation and psychosocial stimulation in childhood with cognitive and psychological well-being outcomes in adulthood.

**Conclusion:**

Our findings suggest that childhood nutrition interventions have long-lasting effects on cognitive ability and psychological well-being outcomes.

## Highlights

• We assess the influence of early-life nutrition on neurodevelopmental outcomes.
• We use a theoretical approach to examine mediators of the association.
• We found no mediators of the association of early-life nutrition with adult psychological well-being.
• Psychosocial stimulation is strongly associated with neurodevelopmental outcomes.
• Improved child nutrition is positively associated with adult psychological well-being.

## 1 Introduction

The period from conception to age two years, often referred to as “the first 1000 days,” is critical for the growth and development of motor, cognitive, and socioemotional skills (Thompson and Nelson, 2001; Victora et al., 2010). Adequate experiences and environments, including prenatal and postnatal nutrition, psychosocial stimulation, characteristics of the physical environment, and maternal mental health during this sensitive period, play a crucial role in optimal brain and child development (Aboud and Yousafzai, 2015).

Observational studies in low-and middle-income countries (LMICs) consistently show that nutritional deficits in the first 1000 days result in impaired growth and cognitive development with long-lasting effects on social and human capital attainment (Victora et al., 2008). Furthermore, randomized trials of food supplements provided during critical age periods to undernourished children living in poverty suggest causal links between child undernutrition, motor and mental development (Grantham-McGregor et al., 1991; Pollitt et al., 2000; Prado et al., 2017), and later cognitive abilities (Maluccio et al., 2009; Pollitt et al., 1993).

Associations between child undernutrition and cognitive abilities are well documented, yet, the socioemotional consequences of early-life nutritional deficits are less well understood. The development of socioemotional skills occurs along with sensory-motor, cognitive, and language capacities and provides the basis for emotional well-being and adult socioemotional functioning and behavior (National Scientific Council on the Developing Child, 2004).

Longitudinal studies examining associations between nutritional status and socioemotional outcomes show that children who were stunted or underweight in early childhood show poorer social skills and more conduct problems at school age than children of adequate nutritional status (Chang et al., 2002; Galler and Ramsey, 1989; Richardson et al., 1972). Only a few nutritional supplementation trials have examined long-term effects on socioemotional outcomes (Barrett and Radke-Yarrow, 1985; Barrett et al., 1982; Chang et al., 2002), and these have follow-up periods only through age eight years. Two of these studies (Barrett and Radke-Yarrow, 1985; Barrett et al., 1982) were conducted in a subsample of school-age children who, in early childhood, participated in a community-randomized food supplementation trial in eastern Guatemala. Children were followed up at ages six to eight years and examined in free-play peer interactions, including problem-solving activities, competitive games, and impulse-control situations. Children who received higher caloric supplementation prenatally (through maternal caloric supplementation during pregnancy), and from birth to age two years, showed more social involvement and interest in the environment and were capable of showing more positive affective expression (Barrett et al., 1982). Furthermore, these children were rated as more energetic and self-confident and were less frequently timid or anxious than children who received less supplementation (Barrett and Radke-Yarrow, 1985; Barrett et al., 1982).

The objective of the present study is twofold. First, we use information from participants of this community-randomized food supplementation trial with new data collected between 2017 and 2019 to examine associations of exposure to nutritional supplementation in early childhood with executive function and psychological well-being at ages 40–57 years. Second, using data collected in this population between 1969-1977 and 2002–2004, we examine the mediating role of psychosocial stimulation in childhood, cognitive ability at ages 26–42 years, and executive function at ages 40–57 years on the association between exposure to nutritional supplementation in early childhood and psychological well-being at ages 40–57 years.

## 2 Background

### 2.1 INCAP longitudinal trial

In the late 1960s, protein deficiency was the major nutritional problem among the poor in developing countries and there was concern that this deficiency could affect the children's ability to learn through the inhibition of brain growth and myelination during critical periods of neural development. The US National Institute of Child Health and Human Development in conjunction with the Institute of Nutrition of Central America and Panama (INCAP) implemented a community-randomized food supplementation trial between 1969 and 1977 in four rural communities in eastern Guatemala. The principal hypothesis motivating the intervention was that improved nutrition would accelerate mental development in children. Two-pairs of villages, matched by population size, were chosen from a pool of 300 communities. Matched villages had similar health indicators and comparable socioeconomic characteristics. At the time of the study design, malnutrition and infectious diseases were endemic in all villages, average family incomes were very low (<$300 US per year), and most adults were illiterate (Bergeron, 1992).

One village from each pair was randomly selected to receive a nutritious supplement called *“atole”* and the other two villages received a control drink called *“fresco”* (Habicht and Martorell, 1992). *Atole* was a moderate-protein and energy gruel-type beverage that contained 11.5 g of protein and 163 kcal per 180 mL serving. *Fresco* was a low-energy drink (59 kcal per 180 mL serving) that contained no protein. Both drinks contained equal concentrations of micronutrients. Each supplement was provided to all residents of the village throughout the study in a supplementation center twice a day, in mid-morning and mid-afternoon. Attendance was open to all residents and was recorded for pregnant and breastfeeding women and children up to age seven years (Habicht and Martorell, 1992; Martorell et al., 1995). Children under seven years were recruited at study inception, as were all children born during the study implementation phase. The final sample consisted of 2392 individuals, all born between 1962 and 1977. The nutritional trial resulted in increased linear growth rates in children exposed to *atole* compared with *fresco* among children ≤ 3 y of age (Schroeder et al., 1995).

The cohort has been surveyed periodically. Data for this paper come from the 2002–2004 and 2017–2019 waves. Both follow-up assessments aimed to examine the contributions of early-life nutrition and other circumstances on adult social and human capital attainment.

### 2.2 Hypotheses for pathways of interest

The theoretical basis for our analytical approach derives from models of nutrition and social behavior and studies suggesting that early-life nutritional deficits compromise the child's ability to engage with the environment and respond to stimulation (Barrett, 1986; Grantham-McGregor et al., 1989; Sigman and Wachs, 1991; Wachs et al., 1992). Additionally, it has been proposed that undernourished children are often more irritable and withdrawn, causing caregivers to treat them with less sensitivity, resulting in altered patterns of brain development (Prado and Dewey, 2014). We further enrich our conceptual framework with evidence from studies indicating that executive function is crucial for all forms of cognitive performance and psychological well-being. Executive function, often considered a domain of cognitive function, is a broad term used to describe a set of higher-order cognitive processes that regulate various cognitive functions, including thoughts and behaviors for the attainment of goals (Diamond, 2013).

**Fig. 1:**
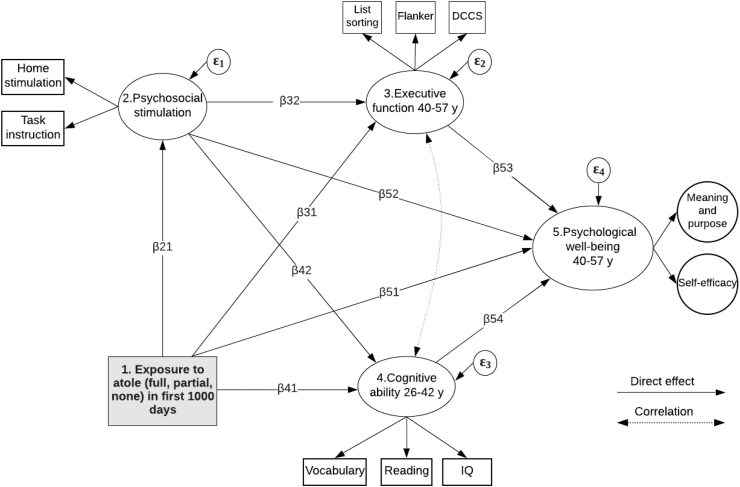
Hypothesized SEM model. Measures for domains 1 and 2 were collected in early childhood, domains 3 and 5 at ages 40–57 years, and domain 4 at ages 26–42 years. Abbreviations: DCCS Dimensional Change Card Sort; IQ Intelligence Quotient.

Fig. 1 outlines our pathways of interest. We hypothesize that children who were exposed to *atole* in the first 1000 days were less irritable or withdrawn, and more engaged with the environment. As a result, these children received more psychosocial stimulation (β_21_). Previous studies in this population have documented positive associations between early-life exposure to *atole* and higher scores in reading comprehension and IQ tests at ages 26–42 years (β_41_) (Maluccio et al., 2009). In this study, we examine whether exposure to *atole* in the first 1000 days is positively associated with executive function (β_31_) and psychological well-being (β_51_) at ages 40–57 years. Studies conducted among institutionalized children have documented associations between early-life psychosocial deprivation and deficits in executive function (β_32_) (Bos et al., 2009; Colvert et al., 2008). Pathways β_42_ and β_52_ are supported by evidence from a study in Jamaica documenting that a psychosocial stimulation intervention provided to stunted children up to age two years resulted in higher cognitive performance and fewer symptoms of depression, social inhibition, and reductions in violent behavior at age 22 years (Walker et al., 2011). Pathways β_53_ and β_54_ are supported by studies showing that various aspects of psychological well-being are influenced by executive function (Luerssen and Ayduk, 2017; Moffitt et al., 2011; Schmeichel et al., 2008), and other non-executive cognitive abilities (Bain et al., 2003; Wigtil and Henriques, 2015), respectively.

## 3 Methods

### 3.1 Study population

Data for this analysis come from participants of the INCAP longitudinal trial with data collected between 1969 and 1977, 2002–2004, and 2017–2019. Detailed information on coverage and attrition by 2002 is provided elsewhere (Grajeda et al., 2005). By 2017, of the original sample of 2392 participants, 385 had died (most from infectious diseases in early childhood), 255 had migrated abroad, and 109 were untraceable, resulting in 1643 who were presumed alive and living in Guatemala and were eligible for enrollment. Of these, 114 could not be contacted after multiple attempts and 261 declined to participate, resulting in 1268 cohort members who provided informed consent (Supplemental Figure 1). Characteristics of participants who were lost to follow-up and those who participated in the 2017–2019 follow-up are presented in Supplemental Table 1.

Ethical approval for the study procedures was obtained by the Institutional Review Boards of Emory University in Atlanta-GA, and the Institute of Nutrition of Central America and Panama (INCAP) in Guatemala City-Guatemala. Written participant consent to participate in the study was obtained at each study wave.

### 3.2 Early life measures (1969–1977)

#### 3.2.1 Exposure to nutritional supplementation

When the trial was launched in 1969, all children up to age seven years and those born between 1969 and 1977 were recruited and followed-up until they reached age seven years or until the trial ended. Thus, the age and periods at which children were exposed to the intervention varied across participants. Our primary interest was to capture exposure to nutritional supplementation in the first 1000 days, that is from conception to age two years. Exposure occurred via maternal supplementation during pregnancy and lactation, and from the child's own consumption before age two years. We are unable to differentiate maternal and child-level supplementation given the design of the original study. We characterized exposure to supplementation based on the child's date of birth (assuming a gestation of 266 days) and the dates during which *atole* or *fresco* supplementation was offered in the villages. Supplementation was initiated on January 1, 1969, and May 1, 1969, in the large and small villages, respectively, and continued until February 28th, 1977 in all villages. Participants born between Sept 24, 1969, and Feb 28, 1975, in the two large villages and those born between Jan 22, 1970, and Feb 28, 1975, in the two small villages were considered exposed from conception to age two years. Children born before Sept 24, 1969, in the two large villages and those born before Jan 22, 1970, in the two small villages were considered to not have been exposed during the full gestation period, whereas those born after Feb 28, 1975, were not exposed up to age two years. Based on these dates, a participant exposed for some of the first 1000 days was categorized as partially exposed to nutritional supplementation in the first 1000 days. We created a dummy variable for exposure to the nutritional supplementation using three levels: 1) fully exposed in the first 1000 days; 2) partially exposed in the first 1000 days; 3) not exposed in the first 1000 days. For sensitivity analysis, we also computed a two-level exposure variable: 1) fully exposed to nutritional supplementation in the first 1000 days; and 2) partially or not exposed to nutritional supplementation in the first 1000 days.

#### 3.2.2 Psychosocial stimulation

We retrieved archived data on social and intellectual stimulation received by participants in their home environments during early childhood. Data on home stimulation was assessed between 1974 and 1975, through direct observation of the number of toys, pictures, books, and other stimulating material objects in the child's home. It also included an interview with mothers assessing child-play practices and interaction with caregivers. A composite score of these measures was available for analysis.

An indicator denominated “task instruction” was selected by the psychologists in the project as a measure of the parents' efforts to provide adult modeling and purposeful learning opportunities. Data on task instruction were obtained from repeated annual interviews conducted between 1969 and 1975 and included parent reports of the interaction of caregivers and siblings with the child in three activities: teaching the child how to count, how to do a household task, and accompanying the child to a nearby town. A composite score of repeated measures collected between 1969 and 1975 was available for analysis.

#### 3.2.3 Potentially confounding variables

During the original trial, information on maternal characteristics, including completed grades of schooling and age at child's birth was obtained by interview. Maternal height was measured to 0.1 cm using standardized protocols. Information on socioeconomic status (SES) was obtained from the village census conducted in 1967 and 1975 of household characteristics and consumer durable goods measured in participant households. A single combined score was created using principal components analyses (Maluccio et al., 2005). The 1967 index score was used for participants born before January 1971, and the 1975 index score for those born from January 1971 onwards.

### 3.3 Adult measures (2002–2004)

A comprehensive assessment of educational attainment was obtained by interview, including the highest grade of formal and informal education completed. Tests of intelligence were assessed with the Raven's Progressive Matrices test (sets A, B, and C), a non-verbal assessment of reasoning ability (Raven et al., 1988). Additionally, all literate participants were administered the Interamerican Series test of Reading Comprehension and Vocabulary. We calculated final scores as the sum of correct responses for each test.

### 3.4 Adult measures (2017–2019)

#### 3.4.1 Executive function

We assessed three core executive functions (working memory, inhibitory control, and cognitive flexibility) using computerized Spanish-version tests from the cognition battery of the NIH Toolbox (Zelazo et al., 2013). A complete description of these tests, including their validation, adaptation for Guatemala, and training for their administration, are provided elsewhere (Wray et al., 2020).

*Working memory* was assessed using the List Sorting Working Memory Test, consisting of a series of objects (i.e., animal or food items) that are visually and auditorily presented on a screen one at a time. In a series of trials, participants are asked to memorize and repeat the information presented to them. We computed a final score as the sum of total items correct across trials (Zelazo et al., 2014).

*Inhibitory control* was measured using the Flanker Inhibitory Control and Attention Test. The task requires participants to indicate the left-right direction of a centrally presented arrow while inhibiting attention to two flankers on either side that surrounds it. On congruent trials, the orientation of the flanking stimuli matches the orientation of the central stimulus, and on incongruent trials, they face the opposite direction (Zelazo et al., 2014).

*Cognitive flexibility* was measured with the Dimensional Change Card Sort (DCCS) test. Participants were presented with two figures and were asked to switch between matching pictures by color and matching pictures by shape (Zelazo et al., 2013).

For both Flanker and DCCS tests, we used the NIH Toolbox cognition composite scores, which uses an algorithm that combines accuracy and reaction time (Heaton et al., 2014).

#### 3.4.2 Psychological well-being

Psychological well-being refers to a person's cognitive and affective evaluation of his or her life which includes the subjective assessment of experiences associated with meaning and self-realization (Ryan and Deci, 2001). We assessed meaning and purpose in life using the NIH Toolbox Meaning and Purpose scale consisting of 9-items rated on a 5-point Likert scale ranging from 1 (strongly disagree) to 5 (strongly agree) (Salsman et al., 2014)*.*

Perceived self-efficacy has been identified as an important psychological resource for maintaining psychological well-being in individuals and is described as an individual's conviction concerning their personal ability to effectively cope with adversities in various areas of social functioning and to the achievement of specific tasks (Bandura, 1997). We measured self-efficacy using the NIH Toolbox Self-efficacy scale (Schwarzer and Jerusalem, 1995). Participants rated 10-items on a 5-point Likert scale ranging from 1 (never) to 5 (very often) (Salsman et al., 2013). We calculated the final scores for each scale as the sum of the item scores on that scale. For all scales with missing items (<1%), we applied a two-way imputation approach (Bernaards and Sijtsma, 2000).

### 3.5 Sample for analysis

Our main analytical sample included cohort participants with available measures in 1969–1977, 2002–2004, and 2017–2019, excluding those with missing values on potentially confounding covariates (*n* = 1640). In some analyses, we further restricted the sample to those with at least one outcome measure during the most recent follow-up (*n* = 1268).

### 3.6 Statistical analysis

#### 3.6.1 Main effects model

We conducted an intention-to-treat analysis to investigate associations of full and partial exposure to nutritional supplementation in the first 1000 days with adult executive function, meaning, and purpose, and self-efficacy using linear regression models with double-difference (DD) estimators controlling for potentially confounding variables. The DD-estimator captures the differential effect of the intervention in children exposed to *atole* relative to *fresco* during critical windows of exposure. The first difference compares the average change in outcome across children exposed to *atole* in the first 1000 days relative to *fresco*. The second difference subtracts the average change in outcome across village treatments outside the first 1000 days window. The interaction term between treatment assignment and age of exposure to supplementation represents the differential effect of full or partial exposure to *atole* in the first 1000 days (DD-estimator). Models included the DD-estimator, dummy variables of study villages to control for village fixed effects, a categorical variable for the age of exposure to the intervention, birth year to control for the age range of the study sample, SES in 1969–1977, maternal grades of schooling, the logarithm of maternal age at child's birth and maternal height (Maluccio et al., 2009; Stein et al., 2008). Because some children were siblings, we accounted for clustering at the household level. We used multiple imputation techniques for missing values on covariates. The prevalence of missing information was 21.3% for maternal height, 3.5% for maternal grades of schooling, and 0.7% for maternal age at respondents' birth. We conducted most of the analyses in Stata 15, and we used Mplus 8 for models assessing latent executive function.

#### 3.6.2 Mediation model

We used structural equation modeling (SEM) to examine the mediating role of psychosocial stimulation and cognitive abilities on the association between exposure to nutritional supplementation in the first 1000 days and psychological well-being in adulthood. An SEM model is a two-part model consisting of a confirmatory factor analysis or measurement model, followed by a structural model. The measurement model assesses factor loadings while the structural model examines associations between factors and observed variables. The model included dummy variables of the interaction term between treatment assignment and age of exposure as the exogenous variable using three levels: 1) exposure to *atole* during the full first 1000 days; 2) exposure to *atole* during a partial period of the first 1000 days; and 3) no exposure to *atole* during the first 1000 days as the reference category. We also conducted sensitivity analyses using a two-level exposure variable: 1) exposure to *atole* during the full first 1000 days; and 2) partial or no exposure to *atole* during the first 1000 days as the reference category. We modeled computed scores on “home stimulation” and “task instruction” as a latent construct called “psychosocial stimulation.”. Scores on Reading comprehension, Vocabulary, and Raven's Progressive Matrices Tests (IQ) were modeled as a latent construct called “cognitive ability at age 26–42 y” List Sorting Working Memory, Flanker Inhibitory Control and Attention, and DCCS Tests scores, were modeled as a latent construct called “executive function at age 40–57 y”. Individual Likert-item responses for Meaning and Purpose and Self-efficacy scales were modeled as latent sub-domains of “psychological well-being at age 40–57 y”. Additionally, in separate models, we tested the joint effects of exposure to *atole* during the full first 1000 days and psychosocial stimulation on executive function, meaning and purpose and self-efficacy using interaction terms.

All paths controlled for village-level fixed effects, SES in 1967–1975, sex, maternal grades of schooling, maternal age at respondents' birth and maternal height (log-transformed), a categorical variable for the age of exposure to the intervention, birth year, and confidence intervals accounted for clustering of subjects within the family. We estimated standardized direct associations, total indirect associations, and total associations for each pathway.

We interpreted the adequacy of model fit with three commonly reported indices. The comparative fit index (CFI), the Tucker-Lewis index (TLI), and the Root Mean Square Error of approximation (RMSEA) (Bentler, 1990; Steiger, 2000; Tucker and Lewis, 1973). CFI >0.90 for good fit; TLI >0.95 for good fit; and RMSEA <0.08 for acceptable fit and <0.05 for good fit. If the model fit was below the threshold for a good fit, we examined modification indices to improve model fit, and if proposed pathways or covariances between variables were justifiable, we re-specified the model.

All SEM models were estimated using MPLUS 8 using the WLSMV estimator for categorical/ordinal responses (Muthén and Muthén, 1998), with pairwise deletion for missing values. Interactions between the psychosocial stimulation latent variable and exposure to nutritional supplementation were estimated using the XWITH command in MPLUS 8.

## 4 Results

Descriptive statistics of the study population are presented in Table 1. Partial exposure to *atole* during the first 1000 days (vs. no exposure) was positively associated with List Sorting working memory scores (β = 1.24 95% CI, 0.05, 2.42), and the executive function latent construct (β = 0.96 95% CI, 0.12, 1.79). Full exposure to *atole* in the first 1000 days (vs. no exposure) was associated with a 1.51-point increment in meaning and purpose scores (95% CI, 0.33, 2.69) (Table 2).

**Table 1:** Descriptive statistics (Mean ± SD or %) of the study population, by exposure to *Atole* in the first 1000 days.

	*n*	Full exposure	*n*	Partial exposure	*n*	No exposure
1969-77 trial						

Males (%)	360	47.8	292	47.9	988	48.0
Maternal schooling (years)	358	1.2 ± 1.5	285	1.3 ± 1.6	946	1.4 ± 1.7
Maternal age at childbirth (years)	358	27.2 ± 7.2	289	26.9 ± 6.9	979	27.0 ± 7.1
Maternal height (cm)	301	148.7 ± 5.3	233	148.7 ± 5.1	750	148.3 ± 5.0
Socioeconomic status (%)	360		292		988	
Low		26.4		28.4		37.4
Medium		35.3		35.3		32.4
High		38.3		36.3		30.2
Home stimulation score^a^	233	−2.27 ± 22.8	150	−3.57 ± 22.6	650	−1.86 ± 28.1
Task instruction score^b^	274	0.29 ± 7.9	202	0.74 ± 8.0	845	0.60 ± 7.8

2002-04 follow-up						

Reading comprehension score (out of 40)	271	23.9 ± 7.1	213	24.3 ± 6.7	713	23.3 ± 6.5
Vocabulary score (out of 45)	271	20.5 ± 10.8	213	20.7 ± 10.2	713	19.7 ± 10.1
Raven Progressive Matrices score (out of 36)	329	18.9 ± 6.3	259	18.5 ± 5.9	864	17.0 ± 6.0

2017-19 follow-up						

Age (years)	273	45.6 ± 1.6	228	45.6 ± 4.0	770	48.6 ± 4.4
Schooling (years)	353	4.5 ± 3.7	290	4.5 ± 3.5	975	5.3 ± 3.6
List Sorting Working Memory score (out of 26)	261	11.9 ± 3.9	217	12.6 ± 3.6	733	11.7 ± 4.0
Flanker Inhibitory Control and Attention score, (NIH score)^c^	264	5.7 ± 1.2	221	5.8 ± 1.1	728	5.4 ± 1.2
DCCS score, (NIH score)^c^	266	5.5 ± 1.9	217	5.4 ± 2.0	740	5.1 ± 1.9
Meaning and Purpose score (out of 45)	266	37.0 ± 4.2	225	36.6 ± 4.0	759	36.5 ± 4.1
Self-efficacy score (out of 40)	265	31.3 ± 6.2	225	30.5 ± 6.6	760	31.2 ± 7.0

Abbreviations: DCCS Dimensional Change Card Sort; NIH National Institutes of Health.

a Computed scores range from −51 to 96.

b Computed scores range from −20 to 20.

c Computed scores range from 0 to 10, but if the score is between 0 and 5, it indicates that the participant did not score high enough in accuracy (80% correct or less).

**Table 2:** Difference-in-difference estimates of full and partial exposure to nutritional supplementation in first 1000 days on executive function, meaning and purpose and self-efficacy.^a^

	Full exposure to *atole* in first 1000 d vs. no exposure	Partial exposure to *atole* in first 1000 d vs. no exposure
Executive function		
List Sorting Working Memory score	0.34 (−0.76, 1.44)	1.24 (0.05, 2.42) *
Flanker NIH score^b^	0.07 (−0.23, 0.38)	0.29 (−0.03, 0.61)
DCCS NIH score^c^	0.22 (−0.37, 0.80)	0.29 (−0.29, 0.87)
Latent executive function	0.37 (−0.61, 1.35)	0.96 (0.12, 1.79) **
Psychological well-being		
Meaning and Purpose score	1.51 (0.33, 2.69) *	0.67 (−0.59, 1.94)
Self-efficacy score	1.87 (−0.08, 3.82)	0.76 (−1.21, 2.74)

a Sample sizes were 1211 for List Sorting Working Memory, 1213 for Flanker, 1223 for DCCS, 1244 for executive function latent variable, 1250 for meaning and purpose and self-efficacy. Estimates are linear regression coefficients (95% CI) for the interaction term specifying full and partial exposure to *atole* (relative to *fresco*) in first 1000 d vs. no exposure controlling for sex, age at intervention (partial and full exposure in first 1000 d vs. no exposure), fixed effects of birth village, birth year, household wealth index in 1969–77, maternal years of schooling, logarithm of maternal age and maternal height. For missing covariates, we used Multiple Imputation techniques. Confidence intervals account for clustering at the mother level. *p < 0.05. **p < 0.01.

b NIH National Institutes of Health.

c DCCS Dimensional Change Card Sort.

**Fig. 2:**
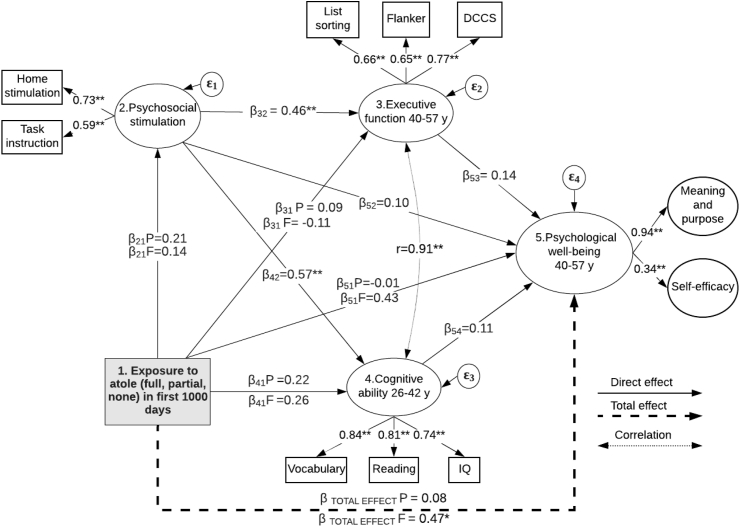
Fitted model of the association between exposure to *atole* in first 1000 d and adult psychological well-being through psychosocial stimulation, executive function, and cognitive ability, (n = 1640). Model fit statistics: RMSEA = 0.02, CFI = 0.99, TLI = 0.98. Estimates are standardized coefficients controlling for: dummy variables for three of the four villages of origin, socioeconomic status in 1967–75, sex, maternal years of schooling, maternal age at respondents' birth and maternal height (log-transformed), age at intervention (partial or full exposure vs. none) and birth year and accounting for clustering of subjects within family. β_21_, β_31_, β_41_, and β_51_ are standardized coefficients (STDY in Mplus 8) for the interaction term specifying full and partial exposure to atole (relative to fresco*)* in first 1000 d vs. no exposure interpreted as the change in *Y* in *Y* standard deviation units when *X* changes from 0 to 1. *p < 0.05, **p < 0.001.

Fig. 2 shows the measurement and structural mediation models for the association of partial or full exposure to nutritional supplementation in the first 1000 days with adult psychological well-being through psychosocial stimulation, cognitive ability, and executive function. The model had good fit (RMSEA = 0.02, CFI = 0.99, TLI = 0.98). We observed significant direct and positive associations of psychosocial stimulation in childhood with executive function at ages 40–57 years (Standardized (STD) β = 0.46, *p <* 0.01), and of psychosocial stimulation with cognitive ability at ages 26–42 years (STD β = 0.57, *p <* 0.01). Furthermore, we found that executive function and cognitive ability were strongly inter-correlated (*r=*0.91, *p <* 0.01).

Table 3 shows standardized coefficients for direct associations, total indirect associations, and total associations for each predictor and dependent variable in our models using a 3-level exposure to nutritional supplementation. We found a significant total association between full exposure to *atole* in the first 1000 days and psychological well-being at ages 40–57 years (STD β = 0.47 95% CI, −0.13, 1.08). Furthermore, we observed positive total associations of full and partial exposure to *atole* in the first 1000 days with cognitive ability at ages 26–42 years (STD β = 0.34 95% CI, −0.12, 0.80; *p=*0.05), and (STD β = 0.34, 95% CI, −0.07, 0.74; *p <* 0.05), respectively. We observed no association between exposure to nutritional supplementation and executive function in the mediation model.

**Table 3:** Standardized direct associations, total indirect associations, and total associations between full and partial exposure to *atole* in first 1000 days and adult psychological well-being through psychosocial stimulation, executive function and cognitive ability, (*n* = 1640).^a^

Dependent variables		Standardized Coefficients (95% CI)		
	Predictors^b^	Direct associations	Total indirect associations	Total associations
Psychosocial stimulation	Full exposure to atole	0.14 (−0.22, 0.50)		
	Partial exposure to atole	0.21 (−0.09, 0.51)		
Executive function 40-57 y	Full exposure to atole	−0.11 (−0.62, 0.39)	0.06 (−0.11, 0.23)	−0.05 (−0.55, 0.45)
	Partial exposure to atole	0.10 (−0.33, 0.53)	0.09 (−0.05, 0.25)	0.19 (−0.23, 0.62)
	Psychosocial stimulation	0.46 (0.20, 0.72) **		
Cognitive ability 26-42 y	Full exposure to atole	0.26 (−0.22, 0.74)	0.08 (−0.13, 0.29)	0.34 (−0.12, 0.80)^c^
	Partial exposure to atole	0.22 (−0.20, 0.63)	0.12 (−0.07, 0.30)	0.34 (−0.07, 0.74) *
	Psychosocial stimulation	0.57 (0.28, 0.85) **		
Psychological well-being 40-57 y	Full exposure to atole	0.43 (−0.22, 1.08)	0.04 (−0.26, 0.35)	0.47 (−0.13, 1.08) *
	Partial exposure to atole	−0.01 (−0.51, 0.49)	0.08 (−0.06, 0.24)	0.07 (−0.42, 0.57)
	Psychosocial stimulation	0.10 (−0.20, 0.40)	0.13 (−0.01, 0.27) *	0.23 (−0.03, 0.50) *
	Executive function	0.14 (−0.60, 0.89)		
	Cognitive ability 26-42 y	0.11 (−0.65, 0.88)		

a Model fit statistics: RMSEA = 0.02, CFI = 0.98, TLI = 0.98. Models controlled for village fixed effects, socioeconomic status in 1967–75, sex, maternal years of schooling, maternal age at respondent's birth and maternal height (log-transformed), age at intervention, birth year, and accounting for clustering of subjects within family. Estimates are standardized coefficients controlling for: dummy variables for three of the four villages of origin, socioeconomic status in 1967–75, sex, maternal years of schooling, maternal age at respondents' birth and maternal height (log-transformed), age at intervention (partial or full exposure vs. no exposure), birth year and accounting for clustering of subjects within family. *P < 0.05, **P < 0.01.

b Coefficients for full or partial exposure to atole are interpreted as the change in *Y* in *Y* standard deviation units when *X* changes from 0 to 1 (STDY in Mplus 8).

c *P* = 0.05.

Results on the association between psychosocial stimulation in childhood and psychological well-being at ages 40–57 years showed a significant total association (STD β = 0.23 95% CI, −0.03, 0.50), and a significant total indirect association (STD β = 0.13 95% CI, −0.01, 0.27). The total association between psychosocial stimulation and adult psychological well-being was smaller in magnitude than that observed between full exposure to *atole* in the first 1000 days and adult psychological well-being (STD β = 0.23 vs. STD β = 0.47, respectively, *p <* 0.05 in both) (Table 3).

Supplemental Table 2 and Supplemental Figure 2 show results from sensitivity analysis using two levels of exposure (full vs. partial and no exposure). Results remained similar to those obtained using three levels of exposure and showed a significant direct association on the relationship between full exposure to *atole* in the first 1000 days and adult psychological well-being (STD β = 0.44 95% CI, −0.02, 0.89).

We observed no interaction between full exposure to *atole* in the first 1000 days and psychosocial stimulation on executive function, cognitive ability, meaning and purpose, and self-efficacy (Supplemental Figures 3-5).

## 5 Discussion

To our knowledge, our study is the first to examine prospectively the effect of an early-childhood nutrition intervention on executive function and psychological well-being in adulthood. Our results indicate that exposure to enhanced nutrition during the first 1000 days had significant benefits on psychological well-being outcomes in adulthood. Furthermore, our findings provide no evidence that early psychosocial stimulation, executive function, or cognitive ability mediate the association between nutritional supplementation in the first 1000 days and adult psychological well-being; rather, our results show significant direct and total associations. These findings are consistent with two previous studies conducted in this cohort documenting better behavioral outcomes at ages six to eight years among children that received higher caloric intakes of nutritional supplementation during infancy (Barrett and Radke-Yarrow, 1985; Barrett et al., 1982). Our results suggest that nutrition interventions have long-lasting effects on psychological well-being outcomes and that benefits acquired in childhood persist throughout adulthood.

The effect sizes for the association of full exposure to *atole* in the first 1000 days, with psychological well-being (STD β = 0.47 95% CI, −0.13, 1.08), and cognitive abilities in adulthood (STD β = 0.34 95% CI, −0.12, 0.80) are larger in magnitude to that of a meta-analysis of nutritional supplementation trials on socio-emotional and cognitive scores among children aged 0–5 years (STD β = 0.07 95% CI, 0.02, 0.12, and STD β = 0.06 95% CI, 0.03, 0.10, respectively) (Prado et al., 2019a). These differences in magnitude could suggest that the benefits of early-life nutritional interventions on neurodevelopmental outcomes may intensify over time. Alternatively, it may also suggest that the duration of nutritional interventions commonly used at ages 0–5 y is too short to observe meaningful effects on neurodevelopmental outcomes, or that the rates of undernutrition found in their control groups is not comparable to that of our study population in 1969–1977.

Furthermore, our results show a total effect of exposure to nutritional supplementation in the first 1000 days with adult cognitive ability (see Table 3). This finding is consistent with previous studies in this population documenting significant benefits of nutritional supplementation from birth to age three years on reading comprehension and intelligence test scores at ages 26–42 years (Maluccio et al., 2009). Our results confirm and extend these previous findings by additionally accounting for measurement error. Our analysis eliminates the specific variance and measurement error by extracting the common variance from multiple measures using confirmatory factor analysis within the structural equation modeling framework.

Moreover, previous studies in this population have documented that exposure to the nutritional intervention increased schooling attainment among women (Maluccio et al., 2009). Therefore, we did not control for schooling in our analysis, because schooling would act as a mediator and not a confounder.

The observed associations of nutritional supplementation in early childhood with neurodevelopmental outcomes in adulthood may involve metabolic pathways. For instance, it has been documented that at least 20% of the body's glucose is used by the infant's brain in a resting state (Raichle, 2010). Thus, low levels of energy in the first few years of life may have the potential to reduce brain activity in sites responsible for mental development. In our study population, the provision of the moderate-energy beverage “*atole”* may have increased the amount of brain activity and development.

An additional explanation for the observed results could involve improvements in childhood linear growth as a result of nutritional supplementation (Schroeder et al., 1995). Linear growth in the first two years of life is one of the strongest correlates of mental development. A recent study in this population examining associations between linear growth trajectories in childhood and neurodevelopmental outcomes in adulthood showed that membership in the high compared with a low trajectory from birth to age three years, and at ages, three to seven years was significantly associated with non-verbal fluid intelligence, working memory, inhibitory control, and cognitive flexibility at ages 40–57 years in men but not women. Similarly, sex-adjusted results showed that membership in the high compared with low trajectory was positively associated with meaning and purpose scores at ages 40–57 years (Ramirez-Luzuriaga et al., 2021).

The association between linear growth and mental development could operate through the acquisition of gross motor skills, allowing the child to access more stimulation through the exploration of the environment (Adolph & Tamis-LeMonda, 2014). Yet, some evidence suggests that the attainment of gross motor skills is independent of variations in linear growth (WHO, 2006), and that age of attainment of motor milestones (i.e., walking and standing alone) is weakly correlated with mental development and IQ at ages 18 and 64 months (Hamadani et al., 2013). Although the existing evidence linking linear growth to mental development supports a strong association (Prado et al., 2019b), it does not support a causal association (Leroy and Frongillo, 2019). Rather, linear growth is thought to be a marker of the inadequacy of the environment in which children develop and may be a proxy for early brain and behavioral development (Aboud and Yousafzai, 2016), remaining intricately intertwined with cognitive and socioemotional domains throughout the life course.

In our study, we did not assess stress hormones in childhood. Yet, another plausible explanation for our results could involve the role of stress on early brain development. It is well documented that during critical periods of brain formation, prolonged exposure to stress hormones can impair the development of neural connections, particularly in regions of the brain dedicated to higher-order skills, causing life-long problems in learning and behavior (Shonkoff et al., 2012). It has been documented that undernutrition exposes the body to stress hormones, specifically cortisol (Kilic et al., 2004; Waterlow, 1992). Moreover, children living in poverty are often exposed to multiple stressors (e, g., domestic or community violence, poor social support, unresponsive parenting) (Blair and Raver, 2016). Thus, early-life exposure to enhanced nutrition may have mitigated the disruptive effects of multiple stressors during critical windows of brain formation.

Another important finding in our study was the strong, positive, and direct associations of psychosocial stimulation in childhood with cognitive ability, executive function, and psychological well-being in adulthood. The total association between psychosocial stimulation and psychological well-being was smaller in magnitude than that observed between exposure to supplementation and psychological well-being. We also found that associations between psychosocial stimulation and cognitive abilities were stronger than those between nutritional supplementation and cognitive abilities (See Table 3). These findings are consistent with current understandings of child development research strongly advocating for the promotion of nurturing and responsive caregiving for children to reach their full developmental potential (Britto et al., 2017). Furthermore, a meta-analysis examining the effects of early-life interventions on child growth and cognitive development found that interventions promoting responsive care and learning opportunities had considerably larger effects on children's cognitive, language, and motor development, than nutritional supplementation interventions (range effect sizes 0.38–48 vs. 0.05–0.08) (Prado et al., 2019a).

The concern that children could be affected by parental exposure to the trial was raised during the design phase of the intervention. The active presence of the team members in the communities, together with home visits to monitor child growth, had the potential to influence social stimulation. Furthermore, daily attendance at the supplementation center could have acted as a social outlet for infants and mothers (Read and Habicht, 1992). These concerns were resolved with the inclusion of a feeding center in control communities. Yet, the possibility that the intervention might have influenced social stimulation in both treatment and control communities cannot be ruled out. Our results indicated no interaction between exposure to *atole* in the first 1000 days and psychosocial stimulation on cognitive and psychological well-being outcomes, suggesting that the observed associations between psychosocial stimulation, cognitive and psychological well-being outcomes acted independently from the nutritional intervention.

Our study had unexpected results. We found positive associations between nutritional supplementation and executive function among participants that were partially rather than fully exposed to *atole* in the first 1000 days. Although the first 1000 days is an important window of opportunity for many cognitive outcomes, the actual developmental processes involved in the acquisition of executive function skills may not follow this exact timeframe. It is well documented that relative to other cognitive processes, executive function skills take the longest to mature (Best and Miller, 2010). More research using different windows of exposure is needed to elucidate the influence of enhanced nutrition in early life on the development of executive function skills.

There are strengths to this study. A major strength was the use of rich and extensive longitudinal data collected over 50 years. Additionally, the methods used for assessing the effects of nutritional supplementation on cognitive and psychological well-being outcomes were well suited for evaluating the intervention, allowing for causal inferences to be more plausible. The use of double difference estimators allowed to isolate the effect of exposure to *atole* in the first 1000 days, relative to the control group *fresco* controlling for village-level differences or other period or cohort effects. This was relevant because treatment assignment was allocated at the village level, and with only two villages per treatment, potential baseline differences between treatment groups may not have been adequately addressed by randomization. Another analytical strength of this study was the use of structural equation modeling techniques. Because latent variables extract the common variance from multiple measures, specific variance and measurement error are largely eliminated, resulting in increased statistical power and more accurate parameter estimates.

### 5.1 Limitations

There are limitations to this study. The main limitation is that comparable measures of executive function and psychological well-being measures were not available at earlier ages, during childhood or adolescence. Yet, this is unlikely to represent a major threat to the validity of our findings, given the substantial stability of executive function skills reported throughout adolescence and early-adulthood (Biederman et al., 2007; Boelema, 2014; Friedman et al., 2016). Another limitation is that psychosocial stimulation was not randomized, so baseline differences that were not fully captured by control variables in our models could be obscuring our results.

Furthermore, our findings may have been limited by attrition. Yet, attrition rates were not differential regarding exposure to *atole* in the first 1000 days, and apart from sex, characteristics of those lost to follow-up were similar to those who participated in the 2017–2019 study. Thus, we did not find evidence to suggest that attrition affected the internal validity of our findings.

## 6 Conclusions

This study fills important gaps by examining the long-term effects of nutrition interventions in a range of neurodevelopmental outcomes in adulthood. We found positive but inconsistent associations of nutritional supplementation in early childhood with executive function and psychological well-being outcomes in adulthood. Psychosocial stimulation, cognitive ability, and executive function did not mediate the association between early-life nutritional supplementation and adult psychological well-being. Moreover, we observed no interaction of exposure to nutritional supplementation and psychosocial stimulation in childhood with cognitive and psychological well-being outcomes in adulthood. We also found strong and positive associations of psychosocial stimulation in childhood with cognitive ability, executive function, and psychological well-being outcomes in adulthood.

Taken together, our findings suggest that in populations in which undernutrition is prevalent, childhood interventions that include nutritional supplementation components may help mitigate the damaging effects of early-life adversity on cognitive and psychological well-being outcomes.

## Credit author statement

ADS designed the research. MJR-L, ADS conceptualized the study. MJR-L conducted the literature search, performed the statistical analysis of the data and wrote the initial manuscript draft. MR-Z led data collection activities. MJR-L, AMD, RM, MR-Z, RW and ADS interpreted findings, contributed to the intellectual content of the work, edited subsequent drafts, read and approved the final manuscript.

## Funding information

This study was funded by the Bill and Melinda Gates Foundation (OPP1164115).
