# Supplementary material for: Influence of enhanced nutrition and psychosocial stimulation in early childhood on cognitive functioning and psychological well-being in Guatemalan adults
Source: Soc Sci Med. 2021 Apr;275:113810. doi: 10.1016/j.socscimed.2021.113810 (PMC8024786; doi:10.1016/j.socscimed.2021.113810)
Supplement: Multimedia component 1 [file mmc1.docx]

**Supplemental Table 1** Characteristics of participants lost to follow-up. Nutrition Supplementation Trial Longitudinal Cohort, Guatemala, 1969-2019

|  | **Death** | | **International Migration** | | **Did not otherwise participate in 2017-19 study wave** | | **Participated in 2017-19 study wave** | | **Total** | | |  |
| --- | --- | --- | --- | --- | --- | --- | --- | --- | --- | --- | --- | --- |
|  | **n** |  | **n** |  | **n** |  | **n** |  | | **n** |  | |
| Female, % | 385 | 41.6 | 252 | 34.9 | 487 | 43.1 | 1268 | 55.5 | | 2392 | 48.6 | |
| Exosure to Atole in first 1000 d, % | 385 |  | 252 |  | 487 |  | 1268 |  | | 2392 |  | |
| Partial exposure |  | 20.8 |  | 20.2 |  | 16.8 |  | 18.0 | |  | 18.4 | |
| Full exposure |  | 24.7 |  | 25.0 |  | 23.0 |  | 21.4 | |  | 22.6 | |
| Year of birth | 385 | 1971  (1968, 1974) | 252 | 1971  (1967, 1974) | 487 | 1970  (1967, 1973) | 1268 | 1970  (1967, 1974) | | 2392 | 1971  (1967, 1974) | |
| Childhood household SES, SD | 385 | -0.01  (-0.61, 0.52) | 252 | 0.08  (-0.48, 0.73) | 487 | -0.01  (-0.58, 0.52) | 1268 | -0.09  (-0.69, 0.39) | | 2392 | -0.03  (-0.61, 0.52) | |
| Maternal age, y | 378 | 26.0  (21.0, 33,0) | 248 | 25  (21, 32) | 459 | 26  (21, 32) | 1259 | 26  (21, 32) | | 2344 | 26  (21, 32) | |
| Maternal height, cm | 295 | 148  (144, 152) | 187 | 149  (146, 152) | 318 | 149  (146, 152) | 997 | 148  (145, 152) | | 1799 | 148  (145, 152) | |
| Maternal schooling, y | 336 | 0.0  (0.0, 2.0) | 228 | 1.0  (0.0, 2.0) | 382 | 0.0  (0.0, 2.0) | 1223 | 1.0  (0.0, 2.0) | | 2169 | 1.0  (0.0, 2.0) | |

Values presented are medians (25^th^, 75^th^ percentiles) or percents

**Supplemental Table 2** Standardized direct associations, total indirect associations, and total associations between full exposure to atole in first 1,000 days (vs. partial and no exposure) and adult psychological well-being through psychosocial stimulation, executive function and cognitive ability (n=1,640) ^†^

| **Dependent variables** |  | **Standardized Coefficients (95% CI**) | | |
| --- | --- | --- | --- | --- |
|  | **Predictors** ^‡^ | **Direct associations** | **Total indirect associations** | **Total associations** |
| Psychosocial stimulation | Full exposure to atole | 0.05 (-0.17, 0.27) |  |  |
| Executive function  40-57 y | Full exposure to atole | -0.16 (-0.52, 0.18) | 0.02 (-0.08, 0.13) | -0.14 (-0.49, 0.20) |
|  | Psychosocial stimulation | 0.47 (0.20, 0.73) ** |  |  |
| Cognitive ability  26-42 y | Full exposure to atole | 0.10 (-0.25, 0.45) | 0.03 (-0.09, 0.16) | 0.13 (-0.20, 0.46) |
|  | Psychosocial stimulation | 0.57 (0.28, 0.86) ** |  |  |
| Psychological well-being  40-57 y | Full exposure to atole | 0.44 (-0.02, 0.89) * | 0.00 (-0.21, 0.22) | 0.44 (0.02, 0.86) ** |
|  | Psychosocial stimulation | 0.10 (-0.21, 0.39) | 0.13 (-0.00, 0.28) * | 0.23 (-0.04, 0.49) * |
|  | Executive function | 0.13 (-0.64, 0.90) |  |  |
|  | Cognitive ability  26-42 y | 0.13 (-0.66, 0.92) |  |  |

^†^ Model fit statistics: RMSEA=0.02, CFI=0.98, TLI=0.98. Models controlled for village fixed effects, socioeconomic status in 1967-75, sex, maternal years of schooling, maternal age at respondent's birth and maternal height (log-transformed), age at intervention, birth year, and accounting for clustering of subjects within family. Estimates are standardized coefficients controlling for: dummy variables for three of the four villages of origin, socioeconomic status in 1967-75, sex, maternal years of schooling, maternal age at respondents' birth and maternal height (log-transformed), age at intervention (full exposure vs. partial and no exposure), birth year and accounting for clustering of subjects within family. * P<0.05, ** P<0.01.

^‡^ Coefficients for full exposure to atole are interpreted as the change in *Y* in *Y* standard deviation units when *X* changes from 0 to 1 (STDY in Mplus 8).


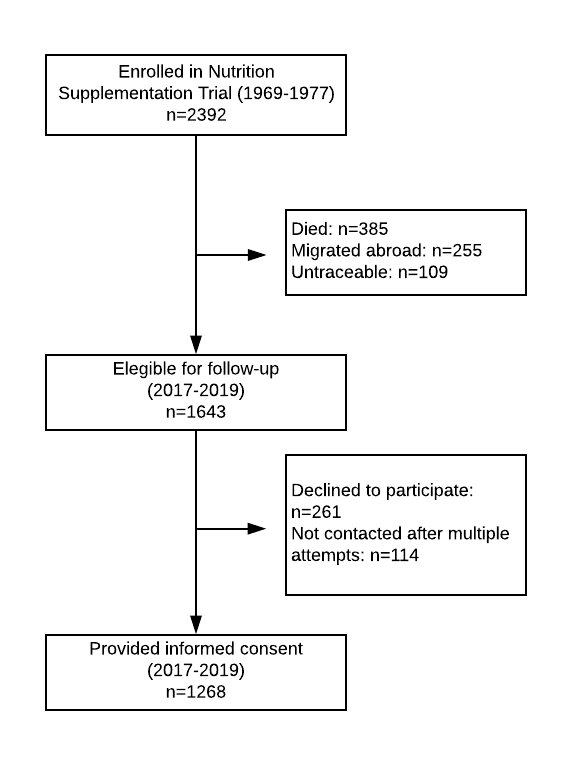


**Supplemental Figure 1** Flowchart of study participants by 2017-19

By 2017, of the original sample of 2,392 participants, 385 had died, 255 had migrated abroad, and 109 were untraceable, resulting in 1,643 who were presumed alive and living in Guatemala and were eligible for enrollment. Of these, 1,268 provided informed consent.


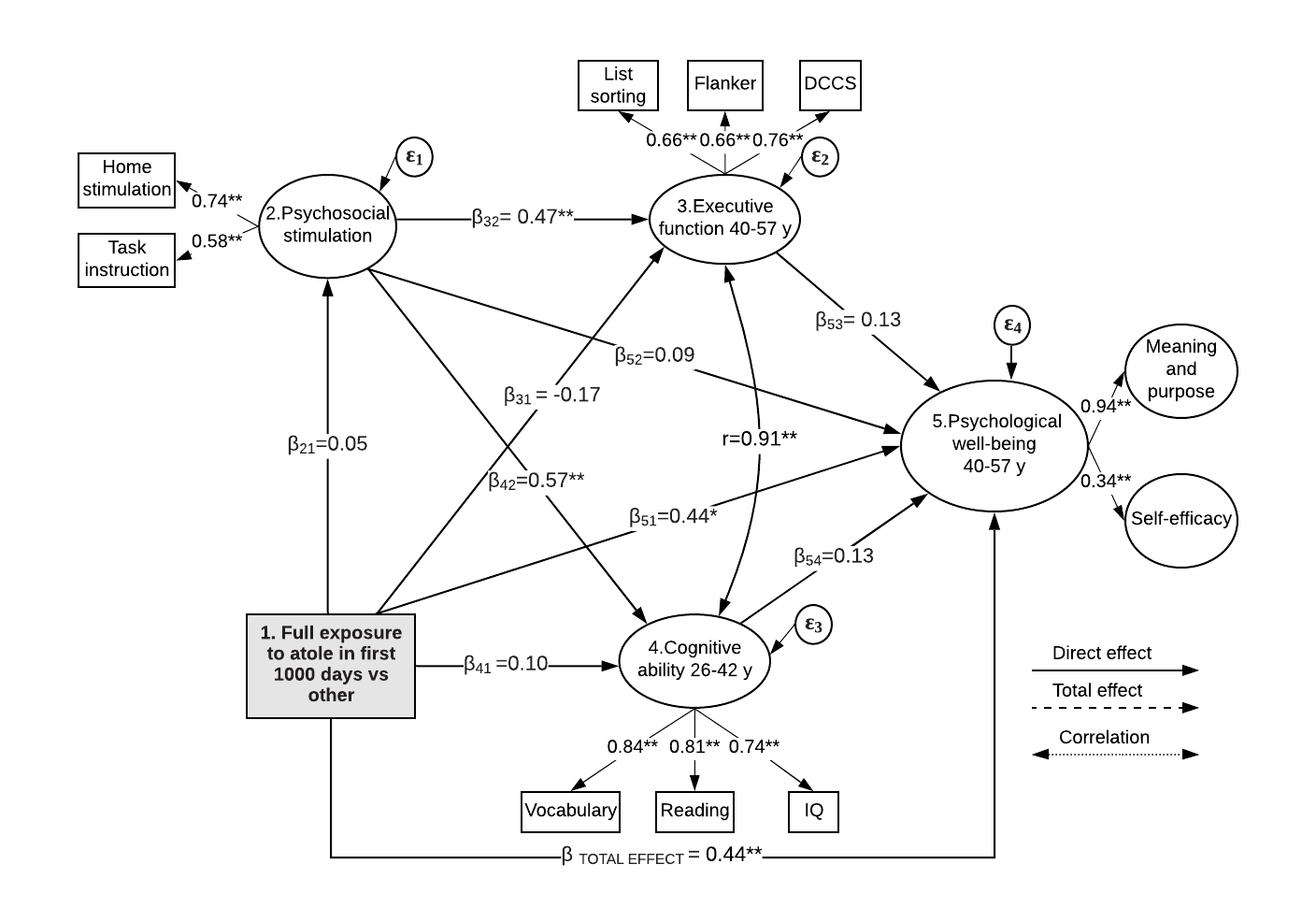


**Supplemental Figure 2** Fitted model of the association between exposure to atole in first 1,000 days and adult psychological well-being through psychosocial stimulation, executive function, and cognitive ability (n=1,640)

Model fit statistics: RMSEA=0.017, CFI=0.98, TLI=0.98. Estimates are standardized coefficients controlling for: dummy variables for three of the four villages of origin, socioeconomic status in 1967-75, sex, maternal years of schooling, maternal age at respondents' birth and maternal height (log-transformed), age at intervention (full exposure vs. other) and birth year and accounting for clustering of subjects within family. β_21_, β_31_, β_41_, and β_51_ are standardized coefficients (STDY in Mplus 8) for the interaction term specifying full exposure to atole in first 1,000 d (vs. other) interpreted as the change in *Y* in *Y* standard deviation units when *X* changes from 0 to 1. *p<0.05, **p<0.01.

**
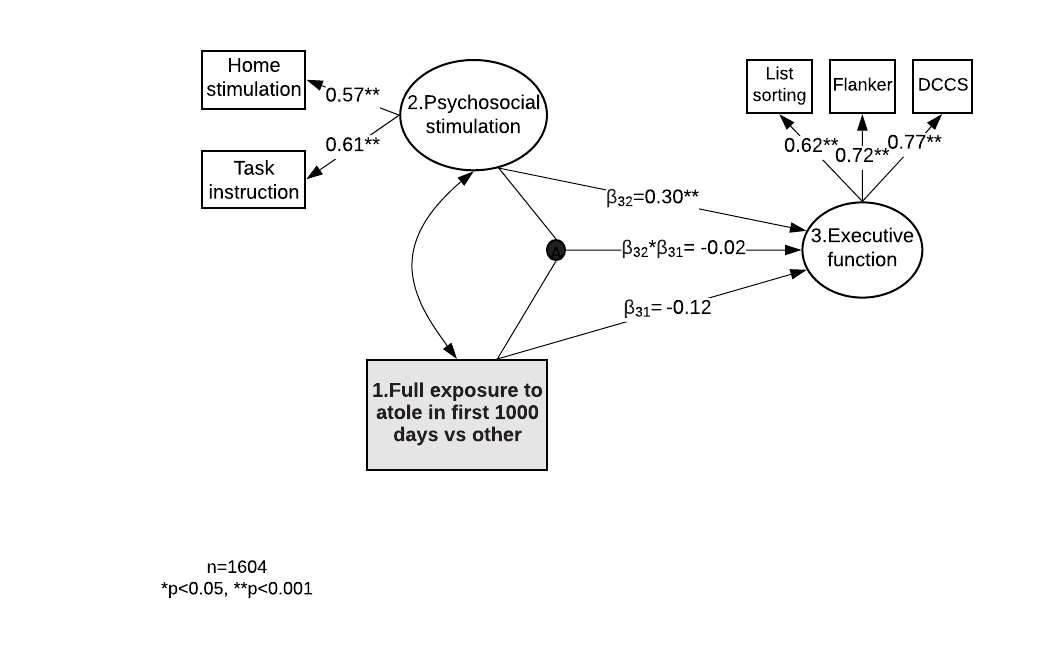
**

**Supplemental Figure 3** Model with interaction between full exposure to atole in the first 1,000 days and psychosocial stimulation on executive function, (n=1604)

Estimates are standardized coefficients controlling for: dummy variables for three of the four villages of origin, socioeconomic status in 1967-75, sex, maternal years of schooling, maternal age at respondents' birth and maternal height (log-transformed), age at intervention (full exposure vs. other) and birth year.


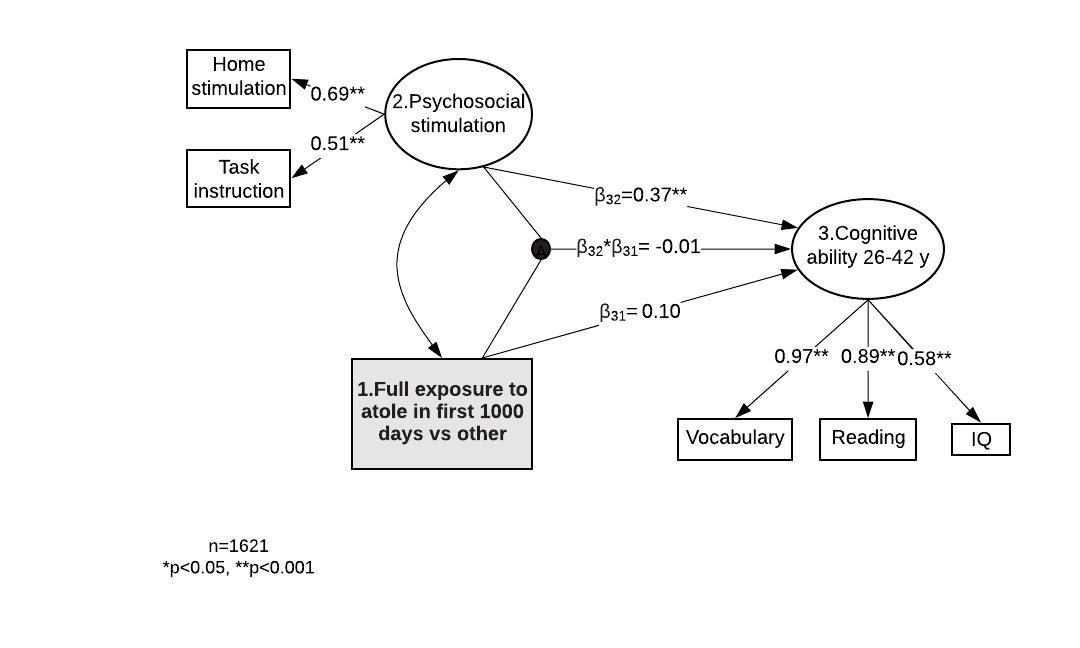


**Supplemental Figure 4** Model with interaction between full exposure to atole in the first 1,000 days and psychosocial stimulation on cognitive ability, (n=1621)

Estimates are standardized coefficients controlling for: dummy variables for three of the four villages of origin, socioeconomic status in 1967-75, sex, maternal years of schooling, maternal age at respondents' birth and maternal height (log-transformed), age at intervention (full exposure vs. other) and birth year.

A)


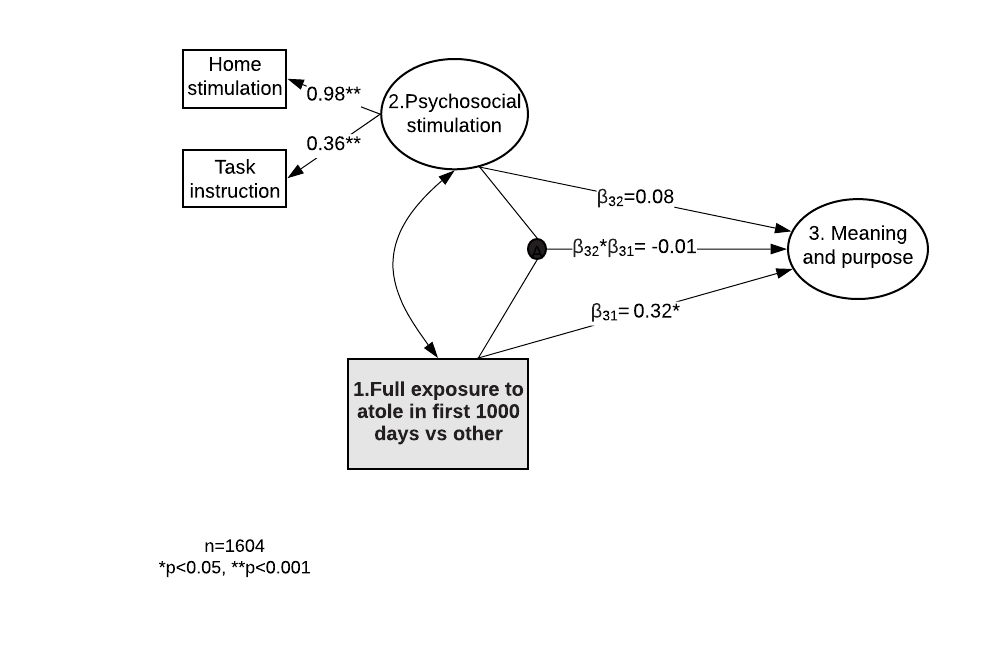


B)


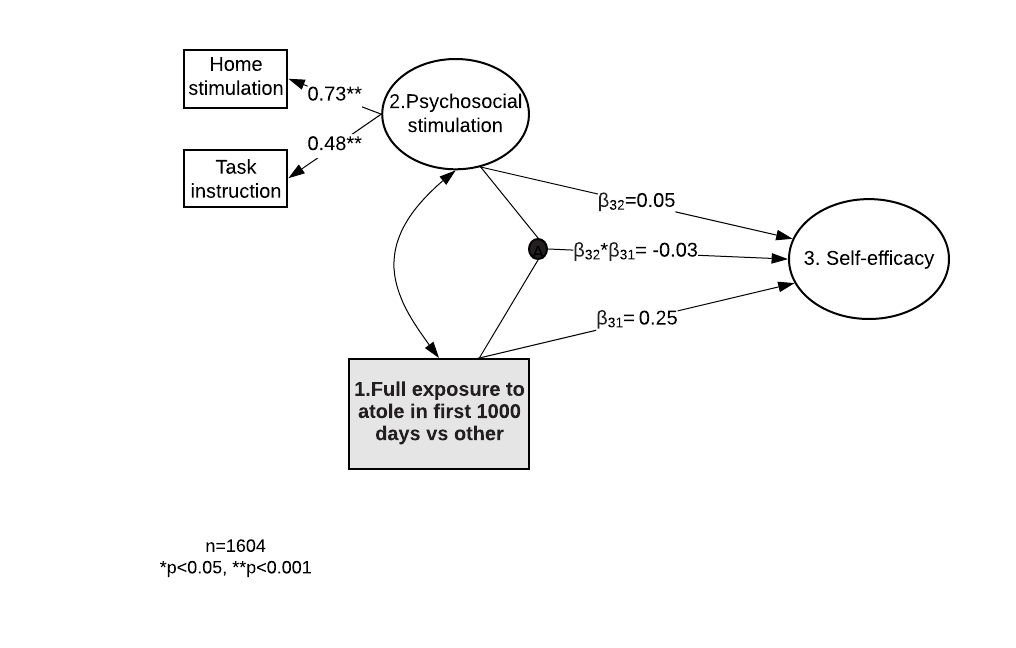


**Supplemental Figure 5** Models with interaction between full exposure to atole in the first 1,000 days and psychosocial stimulation on meaning and purpose (A), and self-efficacy (B), (n=1604)

Estimates are standardized coefficients controlling for: dummy variables for three of the four villages of origin, socioeconomic status in 1967-75, sex, maternal years of schooling, maternal age at respondents' birth and maternal height (log-transformed), age at intervention (full exposure vs. other) and birth year.
